# The influence of prenatal exposure to trans-fatty acids for development of childhood haematopoietic neoplasms (EnTrance): a natural societal experiment and a case-control study

**DOI:** 10.1186/s12937-018-0317-2

**Published:** 2018-01-24

**Authors:** Ina Olmer Specht, Inge Huybrechts, Peder Frederiksen, Eva Steliarova-Foucher, Veronique Chajes, Berit Lilienthal Heitmann

**Affiliations:** 1 Parker Institute, Research Unit for Dietary Studies, Frederiksberg and Bispebjerg Hospital, Frederiksberg, Denmark; 2 International Agency for Research on Cancer (IARC), Nutrition and Metabolism Section, Lyon, France; 3 International Agency for Research on Cancer (IARC), Cancer Surveillance Section (CSU), Lyon, France; 4 Department of Public Health, Section for general Medicine, University of Copenhagen, Copenhagen, Denmark; 5 National Institute of Public health, University of Southern Denmark, Odense, Denmark

**Keywords:** Trans fatty acids, Childhood leukemia, Childhood cancer, In utero, Prenatal, Haematopoietic neoplasms

## Abstract

**Background:**

Little is known about the causes of childhood cancer, partly as not many children develop cancer, although childhood cancer is a leading cause of death by disease in the young. The young age of the children suggests that risk factors for childhood cancer may be present during pregnancy. Previous studies have shown that exposure to trans-fat, a type of unsaturated fat common in industrially produced foods (iTFA), has adverse health effects in adults, including the risk of developing cancer. Haematopoietic neoplasms are the most common cancer types among European children under the age of 15 years. This study will bring new knowledge as to whether trans-fat and other fatty acids may also increase the risk of developing haematopoietic neoplasms during childhood.

**Methods:**

We will investigate if the Danish iTFA legislation ban, which radically reduced the use of iTFA in foodstuffs, influenced the risk of childhood haematopoietic neoplasms in children born either before or after the change in legislation, adjusting for relevant secular trends. Further, in a case-control study, we will examine if levels of fatty acids in dried blood spots from newborns can predict the risk of developing childhood haematopoietic neoplasms. Permission from the Danish Data Protection Agency and the Ethical Committee has been granted.

**Discussion:**

The results from this study will provide important information about fatty acids in the mother’s diet as a contributor to development of haematopoietic neoplasms during childhood, which may result in relevant preventive action.

**Trial registration:**

Not relevant.

## Background

Although childhood cancer is relatively rare, it is a leading cause of death by disease in children under the age of 15 years in Western populations [1], and the incidence is rising [2]. Among the 12 major groups of childhood cancer, leukemia has the highest incidence, with age standardized rate of 49 per million in the age 0–14 years in Northern Europe [3].

The etiology of malignant tumors in the young is mostly unknown. A small proportion of childhood cancers seems inherited [4] or has a viral origin [5, 6]. A few environmental risk factors, such as high-dose radiation and some chemicals are currently recognized as causes of a small proportion of childhood neoplasms [6]. The role of diet in relation to childhood cancers has only sparsely been investigated [7], despite increasing evidence pointing towards diet as a main modifiable risk factor for cancers among adults [8]. Chromosomal translocations noticed in blood samples at birth of children subsequently diagnosed with leukemia, suggest that initiating molecular events in childhood cancer development occur as early as in utero [9]. This emphasizes the need to investigate exposure to potential risk factors already during fetal development. It has been shown that during ‘critical time windows’ of fetal development fetuses are especially vulnerable to environmental exposures. This suggests that lack off, or excess of specific nutritional elements (maternal diet) may program permanent changes in the genome directly by oxidative DNA damage or by epigenetic mechanisms (such as DNA methylation), and in this way modulate later disease risk [10–12].

Trans-fats are a type of unsaturated fatty acids and can be classified as naturally/ruminant (rTFAs) occurring or industrially produced (iTFAs). Industrially produced trans-fats are formed when fats and oils are hydrogenated by industrial processing techniques. The proportions of iTFAs in food are generally much higher than those of rTFAs and in most European countries they are the main dietary source of trans-fats. Examples of foods commonly high in iTFA are baked goods (e.g. pies, biscuits, pastries, sweet rolls) in which shortenings such as margarine are used, and fried foods (e.g. French fries, chicken nuggets), while the counterpart rTFA naturally occurs in low levels in diary and meat products.

Evidence of adverse health effects of iTFAs has been increasing over the past three decades, however rTFA seems to have a less potent effect on cardio metabolic outcomes [13, 14]. The WHO stated that intake of trans-fats should be as low as possible (<1% of total energy intake). Yet intake remains high in most countries worldwide [15–17]. An example of an iTFA high diet could be; one doughnut (3.2 g iTFA), one large portion French fries (6.8 g iTFA) and a bag of microwave popcorn (10 g iTFA), corresponding to overall 23 g iTFA. Guidance to minimize trans-fat intake is based predominantly on evidence that trans-fat consumption significantly increases the risk of coronary heart disease (CHD), where an increase of 2% in total energy derived from iTFA is shown to be associated with an increased risk of death from CHD or myocardial infarction of 23% [14]. In addition, trans-fat intake seems associated with the development of other non-communicable diseases like central adiposity, diabetes, Alzheimer’s disease, endometriosis, cholelithiasis and total mortality [18]. Furthermore, among adults, iTFAs is found to be directly associated with cancer of stomach, multiple myeloma, lung (in never smoking men), mouth/pharynx, breast cancer [19, 20], non-aggressive prostate cancer [21, 22] and colorectal cancer [23].

A case-control study investigating dietary fat and protein intake showed increased risk of non-Hodgkin lymphoma among adults with a diet high in iTFA [24]. TFA has pro-inflammatory properties which might be the mechanism explaining carcinogenesis. A randomized controlled trial in women showed that industrially produced iTFA induced low-grade systemic inflammation by elevating circulating concentrations of tumor necrosis factor (TNF) α and its receptors [25]. TFA exposure in mice during pregnancy and lactation has shown to affect the global DNA methylation in the dams, thus TFA intake may exert long-term effects on the epigenome following maternal exposure [26].

Studies demonstrating relationships between trans-fat intake and cancer in childhood are lacking. However, evidence has shown some possible negative effects on other health outcomes among children. Bouwstra and colleagues have shown that neonates with high TFA umbilical levels have a less favorable neurologic condition at 18 months [27]. Furthermore, a recent review (including human and animal studies) concluded that fetal exposure to trans-fat appears to increase the offspring risk for developing metabolic diseases throughout life [28]. TFAs also interfere in the metabolism of long-chain polyunsaturated fatty acids which might result in a negative impact on infant growth and development [29, 30]. Lifelong, including prenatal, consumption of a diet rich in polyunsaturated fatty acids (PUFA, contained in nuts, seeds and fish) has been shown to protect against tumor growth and to modify glucose metabolism in Walker tumor cells and lymphocytes in Wistar rats [31]. Therefore, the inhibitory effect of PUFA and their interference with trans fatty acids might also be relevant in the fetal programming of childhood cancers.

Despite a general reduction in the iTFA use in food production, Denmark, as the first EU country, legislated against the use of iTFA in all Danish and imported human foodstuffs [32]. The order came into effect on March 31st, 2003, with a transition period until January 1st, 2004. The order imposed a maximum of 2 g of iTFA/100 g oils and fats. During the transition period a maximum level of 5% iTFA was allowed in foodstuffs also containing ingredients other than oils and fats [32]. This legislation led to a radical reduction of iTFA from margarine and shortenings which resulted in a rapid decline in products with trans-fat followed by a decrease in population-level CVD mortality rates [33].

Despite the alarming evidence of increased risk of several diseases caused by TFA consumption and the fact that millions of people still have a too high iTFA intake in Europe, currently no EU legislation regulates the content of iTFA in food products [34].

Given the direct associations found between high TFA intakes and some cancer types among adults, and the deleterious health effects associated with higher TFA intakes among the fetus and offspring, other detrimental effects of an early life exposure to trans-fats, such as childhood cancer, could be envisaged. The health effects of TFA during gestation have never previously been followed in large populations, in part because large-scale interventions are needed to examine how TFA exposure during pregnancy affects long-term health outcomes of offspring, and in particular the risk of developing childhood cancer. However, such interventions may never be conducted due to the associated high financial and logistical costs and ethical aspects. Therefore, alternative study designs should be used to explore associations between early life trans-fat exposure and childhood cancers.

### Hypotheses and objectives

We hypothesize that high TFA exposures, and in particular iTFA exposures, during the critical phase of fetal development increase the risk of developing childhood cancers in general and haematopoietic neoplasms in particular.

We investigate this in two broad designs and study objectives:

1. We will examine if the legislatively reduced use of iTFA in the production of human foodstuff for the Danish market has shown any beneficial effect on population level in preventing childhood haematopoietic neoplasms. Thus we will compare the incidence of childhood haematopoietic neoplasms between all Danish children born in the years before and after the law came into effect. The specific hypothesis is that individuals born in 2004–2008 were exposed to reduced iTFA during fetal development and will have a lower risk of developing childhood haematopoietic neoplasms compared to individuals born in 1988–2003, before the policy was implemented, while taking into account the underlying secular trends in these cancers.
2. We aim to compare neonatal levels of TFA in children with and without a later diagnosis of childhood haematopoietic neoplasm in a matched case-control study. As a secondary aim, we wish to investigate the interactions between TFA and polyunsaturated fatty acids, and their impact on risk of haematopoietic neoplasms in children, since TFA have been shown to inhibit the metabolism of polyunsaturated fatty acids.

The aim of this protocol is to describe the methods and analyses that will be used in this study to investigate the influence of prenatal exposure to trans-fatty acids for development of haematopoietic neoplasms (EnTrance). If deviations from this initial study protocol occur during data analyses, we will update the protocol.

## Methods

As childhood cancers are histologically and etiologically very diverse, investigating effects of prenatal TFA intake on each type of the childhood cancer would require very large sample sizes given the low incidence rates of different tumour types. Therefore, we restrict our study to investigating the potential adverse effects of a high TFA exposure on risk of haematopoietic neoplasms (ICD-10 codes: DC81-C96), the most frequent cancer types in children.

The two objectives of this study reinforce each other by first investigating effects of the trans-fat order on fetal programming of haematopoietic neoplasms on population level and second by investigating variations in biomarkers of TFA and risk of haematopoietic neoplasms, based on measurements of individual exposures. Therefore, null findings in the first objective would not necessarily imply null findings in the second objective.

### Study population

The cancer registry is complete until December 31st 2015. To secure equal time of follow-up for cancer diagnosis in all individuals we censor at 7 years of age, which is the longest follow-up time in which we have information on possible cancer outcomes for all individuals.

We will exclude all children born with a Down’s Syndrome (DS) diagnoses due to their increased risk of childhood leukemia [35] and due to secular trends in DS incidence, because of a new screening practice for DS and other chromosomal aberrations introduced in 2004.

**Fig. 1:**
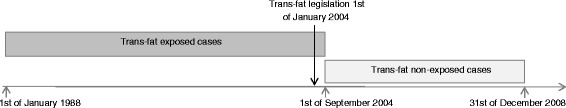
Constitution of the exposed versus non-exposed groups of cases who developed haematological malignancy before the age of 7 years and registered in The Danish Cancer Registry (Objective 1)

*In the first objective,* national birth cohorts of individuals born between January 1st, 1988 and December 31st, 2008 in Denmark will be included (Fig. 1). This is a register based study using the Danish civil registration number which will be linked to the Danish National Cancer Register, where the cancer patients are followed-up until December 31st, 2015.

The incidence of childhood haematopoietic neoplasms among 1,101,055 individuals from the period before the iTFA legislation born between January 1st, 1988 and August 31st, 2004, will be compared to the incidence of childhood haematopoietic neoplasms among 276,422 individuals from the period after the iTFA legislations born between September 1st, 2004 and December 31st, 2008, as a proxy of iTFA gestationally exposed or not exposed, respecitvely. In this overall time period approximately 740 incident haematopoietic neoplasms cases occurred in children before the age of 7 years.

**Fig. 2:**
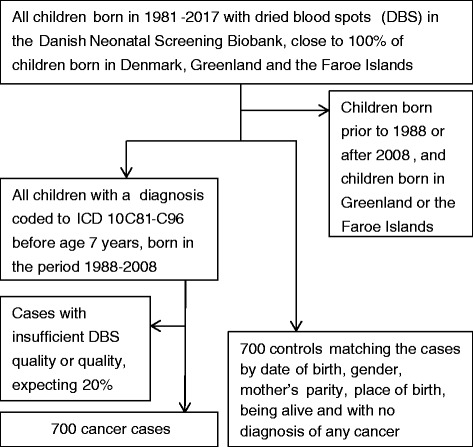
Flow chart of the matched case-control study (Objective 2)

*The second objective* is a case-control study investigating biomarkers of neonatal trans-fatty acid concentrations from stored DBS [34] from individuals who developed childhood haematopoietic neoplasms before the age of 7 years and born in the period 1988–2008 compared to matched controls (Fig. 2).

Since 1981, routinely collected DBS samples taken by heel prick 48–72 h after birth have been collected for all new-born in Denmark. DBS cards are stored in the Biological Specimen Bank for Neonatal Screening at Statens Serum Institute and have been made available to the project [36].

For each case with haematopoietic neoplasms, a control, individually matched for gender, exact date and year of birth, mother’s parity, place of birth, being alive and with no diagnosis of cancer, will be selected and fatty acids isomers measured in the DBS. A case is defined as a child, less than 7 years, with a cancer diagnosis (ICD-10 DC81-C96) and without a DS diagnosis [37]. If more than one participant meets the matching criteria a random control from the eligible participants will be selected. If no participant meets the matching criteria the criteria for exact date of birth or place of birth will be relaxed as needed to match controls to cases and the adjustments made will be reported.

Previous studies have confirmed that storage time of DBS as long as 20 years do not bias inter-individual variation in concentrations of vitamin D and possible other fat soluble substances [38]. Furthermore, as date of birth is used as matching criterion for the controls, potential degradation of TFA would affect equally cases and controls and is therefore unlikely to bias the results.

The methodology for measuring biomarkers of fatty acids in DBS has been set up at the International Agency of Research of Cancer (IARC) in Lyon, France, a specialized WHO and UN organization. The DBS will securely be transported from Denmark to IARC, Lyon, France, where they will be kept in a − 80 °C freezer until analysis. Our validated gas chromatography methodology allows a complete separation, identification and quantification of sixty fatty acids from short-chain saturated to long-chain polyunsaturated fatty acids, including fifteen individual isomers of industrial trans fatty acids (elaidic acid, trans n-6 and n-3 PUFA) separated from natural trans fatty acids (CLA, vaccenic acid) in one-hour runs. The laboratory involved in the set up at IARC is at the cutting edge of lipidomic technologies and have developed sound and innovative high-throughput methods for fatty acid measurements [20]. Fatty acid concentrations will be determined in plasma phospholipids, as some specific fatty acids measured in this lipid fraction have been shown to be reliable biomarkers of specific dietary intakes, as well as fatty acid metabolism [39–41]. Samples from cases and their matched controls will be analysed within the same daily batch, with two quality controls in each batch. The technical staff will not have any information on the case/control status. Total lipids will be extracted from plasma samples with chloroform-methanol containing antioxidant butylated hydroxytoluene and L-A-phosphatidylcholine-dimyristoyl-d as an internal standard. Phospholipids will be purified by adsorption chromatography; fatty acid methyl esters will be separated through gas chromatography. The relative amount of each fatty acid, expressed as percent of total fatty acids, will be quantified by integrating the area under the peak and dividing the result by the total area. Fatty acids will also be expressed as absolute concentrations in plasma (μmol/l) based on the quantity of the methyl deuterated internal standard.

Based on this methodology, coefficients of variation (CVs) for fatty acids have been calculated for 60 samples. CVs ranged from 0.013% for large peaks to 7.75% for the smallest peaks (less than 0.10% of total fatty acids). Overall CVs (within-batch and between batch CVs) are very good for iTFA isomers, mainly for elaidic acid (CV = 0.137%). Overall CV is 0.122% for total iTFA. CVs for rTFAs are also good, mainly for vaccenic acid, the main iTFA isomer (CV = 0.282%). CV for total iTFA is 0.123%.

Because the TFA levels in the new-born blood also reflects the TFA status of the mother at the end of pregnancy, measurement of iTFA levels in DBS will furthermore serve as validation for the effect of trans-fat elimination for incident cases.

To take into account different cohort effects or changes in life-style in the period 1988 to 2008, we will merge the cohort data with information from the Danish Medical Birth Registry on trends in co-variates which might have changed during the time period, e.g. smoking during pregnancy and age of the mother.

### Power calculation

Using the command stpower for the log-rank test for comparison of two survival functions in Stata 14 we calculated the least detectable hazard ratio (exposed versus unexposed) to be 1.35 assuming 80% power and a significance level of 5% in objective 1 (the cohort study) [42].

In objective 2 (the case-control study) we have a sample size of 700 cancer cases and 700 controls, and assuming 80% power, the least detectable OR will be between 1.35 and 1.75, depending on the exposure among the controls (10–90%). The power analysis for the matched 1:1 case-control study was performed using the power mcc command in Stata 14 [43].

### Statistical analysis

In objective 1 we will use a Cox proportional hazard model with age as the underlying timescale to investigate the hazard of childhood haematopoietic neoplasms in children born before and after the iTFA ban, as a proxy for iTFA exposed or not exposed in fetal life. Observations will be censored in case of death, immigration or at 7 years of age. To take the potential secular trend in the incidence of haematopoietic neoplasms into account we will model the variation in cancer risk across birth cohorts by a piecewise linear spline with a knot at 1st of September 2004. Further, we will investigate incidence trends over time.

By examining *all* children from the entire national birth cohorts it is reasonable to assume that all confounding variables will be equally distributed among the exposed and non-exposed individuals, but still we will investigate potential influence of secular variation or possible confounding effects which might have changed during the time period and which might be associated with both TFA exposure and haematopoietic neoplasms. Such potential confounders could be age of the mother at the time of giving birth, assuming accumulation of TFA with age and an older age for first time pregnancies over time. The median gestational age might have increased during the investigated time period, which would increase the average intrauterine exposure to TFA over the study period [44]. Smoking status during pregnancy might have decreased over time and smoking might be associated with increased cancer risk, smoking is also associated with gestational age and thereby with the length of the TFA exposure during pregnancy.

As sub-analysis we will exclude cancer cases diagnosed before the 90 day of life since the origin most likely is due to chromosomal alterations leading to oncogene activation [37]. If feasible we will investigate the first born only since some studies have shown higher risk of childhood cancers in the first born compared to their younger siblings [45] and sensitivity analysis of sub-types of haematopoietic neoplasms (leukemia and lymphoma). Further, we will investigate children with early diagnosis, e.g. before age 4 years.

Conditional logistic regression analyses will be used in the 2nd objective to compare blood spot fatty acid levels between subjects with haematopoietic neoplasms and matched subjects who have not developed cancer. We will investigate industrial as well as ruminant TFA independently and mutually adjusted. Based on a priori consideration we will adjust for maternal and paternal age, smoking during pregnancy, birth weight and gestational age. Also, there might be regional and urban/rural differences in iTFA intake. Place of residence is available from Statistics Denmark, and can be included into analyses. Diets are usually different between families with higher and lower socioeconomic status and maternal socioeconomic status may influence intake of certain food products rich in trans-fat. Information on socioeconomic status, education and ethnicity is available from Statistics Denmark and can be controlled for.

As mentioned in the methods description, other fatty acids potentially related to haematopoietic neoplasms in children will be analysed in the DBS since our methodology allows a complete separation, identification and quantification of sixty fatty acids. We will therefore also investigate interactions between TFA and polyunsaturated fatty acids, and risk of haematopoietic neoplasms in children as well as association between polyunsaturated fatty acids and risk of cancer stratified by trans fatty acids.

## Discussion

Our main goal for this study is to strengthen the scientific basis for understanding idiopathic haematopoietic neoplasms in children. By presenting the protocol before undertaking the actual analyses, we intend to improve the quality, integrity and transparency of the study.

Strengths of the first objective include the unselected national cohort, the prospective design and the register-based measure of outcome. This explorative study takes advantage of the iTFA elimination policy implemented in Denmark almost fifteen years ago, and nests this “societal experiment” in a setting where the effect of conditions from conception (and before) for development of certain diseases can be followed among individuals from birth and onwards. The study is possible due to complete identification of every resident in Denmark via a civil registration number. This number can be linked, on an individual level, to Danish birth, patient and medical registries; social and ethnic registries; and clinical and other national databases. The comprehensive linkage of datasets for both aims makes it possible to study mediation, confounding and/or modification of effects of iTFA for childhood haematopoietic neoplasms.

For the first objective we do not collect any individual data on iTFA intake. In contrast, all individuals are unselected in relation to iTFA exposure and later disease occurrence. This may be considered a very strong feature of the present study, as neither confounding from external or internal factors, nor the lack of individual data on iTFA intake, is a prerequisite for the study, as the influences of lifestyle differences (e.g. use of infant formula, individual diet intake, use of supplements), or biological (adipose tissue stores, weight changes during pregnancy and post-partum) which we would not be able to adjust for, are all independent of the iTFA legislation.

Nevertheless, the proposed study design, especially in the first objective, also implies some uncertainties, including a potential ecological fallacy. The exposure measure is applied equally to all members of the cohort before and after the ban, while the individual exposures have probably differed. However, a previous study has shown a marked decline of average iTFA intake resulting in elimination after the ban [32]. In the case-control study we investigate TFA exposure individually in all cases from 1988 to 2008. The variations in the exposure observed in the case-control study will then inform our assumptions taken in the cohort study.

Any societal changes preceding 1988 or succeeding 2008 will influence the exposed and control groups similarly, while results will be confounded by any potential societal changes occurring around the time where the trans-fat ban was implemented (e.g. 2003/04). However, we are unaware of such changes except for a screening for DS which we have taken into account by excluding children with DS.

Potentially exposure to TFA from maternal adipose stores could affect the “unexposed” group (born after the ban of iTFA) and postnatal influences like maternal feeding practice and weight gain, may also influence cancer risk through a non-TFA mechanism. Although this may indeed seem a possible limitation, since literature has shown that weight loss of the mother (e.g. during lactation) liberates TFA fat deposits [44], proper randomization in our societal experiment study is mimicked by separating exposed and non-exposed individuals by one point in time with a minimum of selection bias. Thus, it is an obvious strength to the present research design, that the individuals will be unselected, and generally representative of the Danish population, in relation to exposed vs. non-exposed to the trans-fat legislation, as well as to the development of haematopoietic neoplasms. However, since we do not know the exact mechanism between TFA and childhood neoplasms, we cannot account for a residual confounding which might affect observed associations. Residual confounding could be caused by additional components in food products with high contents of TFA. We are not able to investigate TFA levels at specific developmental stages during gestation, but since no knowledge exist on specific target windows for childhood cancers, this may not be relevant.

Other health outcomes in children, which will not be described in this protocol, will be investigated in future studies using the same study designs, include: negative birth outcomes, cognitive function, epilepsy, asthma, heart disease, eye diseases, bone fracture, and metabolic diseases like type 1 diabetes and childhood obesity.

## Abbreviations

DBS: Dried blood spots
DS: Down’s syndrome
IARC: The International Agency of Research of Cancer
iTFA: Industrially produced trans fatty acids
PUFA: Polyunsaturated fatty acids
rTFA: Ruminant trans fatty acids
TFA: Trans fatty acids
TNF: Tumor necrosis factor α
